# Exploring Strategies for Improving Green Open Spaces in Old Downtown Residential Communities from the Perspective of Public Health to Enhance the Health and Well-Being of the Aged

**DOI:** 10.1155/2021/5547749

**Published:** 2021-06-14

**Authors:** Si-Jie Li, Yu-Feng Luo, Zi-Chuan Liu, Lei Xiong, Bo-Wei Zhu

**Affiliations:** ^1^Faculty of Humanities and Arts, Macau University of Science and Technology, Avenida Wai Long, Taipa 999078, Macau, China; ^2^Academy of Fine Arts, South China Normal University, Guangzhou 510631, China; ^3^School of Architecture and Allied Art, Guangzhou Academy of Fine Arts, Guangzhou 511400, China

## Abstract

Based on the trend of global aging, people are paying more and more attention to the health of the elderly and the improvement of green open spaces. However, few studies have focused on strategies to improve green spaces in response to this trend. Especially, with the outbreak of COVID-19, an urgent need to develop a sustainable system strategy to improve the health of the elderly in residential communities in old districts has emerged. Traditional improvement strategies based on current situation evaluation often focus on the most prominent practical problems. Therefore, the objective of this study was to provide theoretical research and practical improvement strategies for green open spaces in old downtown residential communities to improve the health and well-being of the elderly. In response to this problem, this research proposes an alternative method based on causality (FDM-DANP-mV model), by extracting 23 green open space elements that affect the health of the elderly and dividing them into three dimensions, to form a preliminary evaluation framework. On this basis, the more effective and feasible standard elements are screened out, and the influence relationship behind the elements is clarified. Then, the sustainable development strategy is systematically discussed in three practical cases. This allows for the analysis of the present situation to not only identify the current significant problems but also to capture the source of the influence behind the real problems based on the clarification of the dominant influence relationship. The actual value of this study is to provide a key design decision basis for the improvement of the green open spaces in old downtown residential communities, aiming at avoiding waste to the greatest extent under the premise of limited resources and gradually promoting the improvement of the urban built environment to promote the health and well-being of the elderly.

## 1. Introduction

With the aging of the global population, the World Health Organization (WHO) has put forward a policy of “healthy aging” [1], with “providing the aged with safe, inclusive and barrier-free public spaces” [2] as the goal of global aging health strategy and action. And, China has become an important representative of healthy aging [3]. When taking public interest into account, incorporating the growing health needs of the aged into the development of public open spaces (POS) has become a major challenge of the 21st century [4]. In addition, as said by the WHO, whether long life will become a social burden depends on the health of the aged to a large extent [2]. At present, the sudden outbreak and wide coverage of COVID-19 brings new challenges to urban planning and architectural design [5]. How to reduce the dissemination of the virus and restore economic growth through optimal urban planning and architectural design has become an urgent topic in the post-epidemic age [6]. Having low autoimmunity and mostly suffering from underlying diseases, the aged are susceptible to the virus and once infected are difficult to cure. As reported, most of the infected and critically ill patients are vulnerable elderly people [7].

As the center of early commercial and cultural activities in many developing countries, most old urban areas have superior geographical location [8], still retain the imprint of the times, and show unique characteristics of the times. Because these communities have been around for long time, a large number of elderly people have long lived in these areas, and the phenomenon of community aging has become serious. Generally, the green space in these communities is small and disordered, chaotic, and the infrastructure is old and in need of maintenance. Thus, the quality of public open space is low, and the social function has been neglected. As such, these spaces cannot meet the demands of the elderly for green open space [9]. The outbreak of the novel coronavirus also had an indirect impact. One survey showed that China's overall emotional health decreased by 74% [10]. Moreover, the majority of elderly people have long been at high risk of experiencing mental health problems [11]. Therefore, people have begun to pay attention to health problems and maintain health by participating in activities [12]. Ekstrom [13] pointed out that the uncertainty of the new environment has caused pressure on the elderly, and resettlement will have a negative impact on their mental health, resulting in increased pressure and loss of social support. Urban renewal design aims to solve the problem of unplanned urban aging and promote the sustainable development of cities, while helping to preserve the identity and sense of belonging of the elderly to the original community, emphasize the inclusiveness of the society, and promote the social participation and active aging of the elderly [14]. As a result, providing a green landscape space for the elderly to enhance health and well-being should not be done simply by reprogramming and updating the full picture [15]. At the same time, the outbreak of the epidemic poses a new challenge to urban and architectural design, which is an important part of the national economy, [16]. Epidemics are more likely to erupt in urban areas, arousing our reflection and new exploration in the direction of epidemic prevention through urban construction [17].

In a previous study, Owen Douglas et al. [18] proposed to sort out and review the relationship between health, happiness, and green open space using the life cycle method. In the whole life cycle, they suggested, the key points of planning green open space that is conducive to enhancing the health and well-being of the aged are as follows: first, providing less-intense sports facilities to encourage social interaction and interaction with nature; second, incorporating spontaneous and leisurely opportunities for interaction with the environment into the design of green open space; third, providing sheltered seating areas, sources of drinking water, and toilets. Taking Iran as an example, Azadeh Lak et al. [19], clarified the preferences of the aged for active aging when using public places, and proposed that social environment, sense of belonging, cultural background, and ultimate life satisfaction were the top-priority factors. Perception and preference of the aged to urban space can be independently understood from each of these factors. Furthermore, the design strategies that can promote the health of the aged include: (1) functional quality (such as safety, aesthetics, convenience, cleanliness, comfort, density and urban landscape, and the putting quality of the place first); (2) preferred quality (such as wayfinding ability, subjective aesthetics, safety and control of the fear of falling, which will make older people prefer open spaces); and (3) an environment that has an active and significant impact on preferences of the aged, especially including the social environment (social interaction and civic participation), cultural environment (cultural and religious beliefs), sense of belonging, and life satisfaction in different contexts. With respect to the disorder of green spaces in old urban areas, Chen et al. [20] put forward the following improvement strategies for the purpose of increasing the humanistic and ecological benefits of street landscapes: (1) strengthening the standardized management of municipal sanitation and engineering construction and paying attention to garbage removal, greening, maintenance, and overall environmental governance in corners; (2) attaching importance to the overall ecology and landscape design of urban areas, providing tree ponds, rain gardens, and other sponge facilities as per the local conditions and taking into full consideration the integration of planting and maintenance; and (3) dynamic monitoring of the environmental greening facilities in appropriate combination with digital sensor techniques to guide smart street management. Meanwhile, street furniture and small works of art can be combined to achieve higher humanistic and ecological benefits and increase the interest and artistry of urban space.

To sum up, it is believed that based on the premise of limited resources, it is necessary to focus on improving the current situations of sites and to carry out adaptive adjustments. Obviously, this needs to base on a systematic evaluation of the current situation of a site. And, the examination of real problems and the formulation of applicable improvement strategies need to be completed in combination with a dynamic influence and complementary viewpoints. This helps to formulate a systematic improvement strategy starting from the root cause based on the actual situation of the site [21–23]. Therefore, it is urgently necessary to construct an evaluation framework for evaluating the current situations of green open spaces in downtown residential communities, and clarify the dominant influence relation between the criteria based on criterion weight training. This is the basis for making subsequent evaluation and analysis of the performance in real cases. The results of performance evaluation should reveal the gap between empirical cases and the desired level. Furthermore, a systematic continuous improvement strategy should be formulated for real cases based on the above evaluation and analysis.

The purpose of this research was to extract the construction criteria of green open spaces in old downtown residential communities based on construction of the health and well-being of the aged under the influence of the outbreak, make clear the priorities of the criteria and the dominant influence relation between the criteria, further evaluate the performance of empirical cases on this basis, and explore sustainable improvement strategies for different empirical cases. Therefore, the process of this research is as follows: first, we extract the criteria relevant to improving green open spaces in old downtown residential communities for enhancing the health and well-being of the aged under the influence of the outbreak and initially establish an index framework system on this basis; second, we screen out the evaluation criteria and judge the stability and effectiveness of the evaluation framework using the Fuzzy Delphi Method (FDM); further, we identify the weight of each index by applying the DANP technique while clarifying the complicated and key dominant influence relations between the criteria; finally, we evaluate and analyse the performance of the empirical cases by using a modified VIKOR technique, making clear the gap between each empirical case and the desired level and further making strategies for improving green open spaces in old downtown residential communities to enhance the health and well-being of the aged. This allows for the proposed situation evaluation and analysis to not only identify the current significant problems but also to capture the source of the influence behind the real problems based on the clarification of the dominant influence relationships, and avoid waste to the greatest extent under the premise of limited resources. Therefore, the practical value of this study is to provide a direction for the transformation of residential areas in the centers of old cities, and to guide researchers and decision-makers in theory and practice on how to enhance the health and well-being of the elderly by improving the real root causes.

The structure of the rest of this paper is as follows: In Section 2, relevant elements are summarized and the research evaluation framework is initially constructed through a review of key previous studies of outdoor activities, connecting with nature, and social interaction; in Section 3, the methods and steps used in this research are introduced in detail; in Section 4, FDM is used to screen the effectiveness of the criteria, DANP is used to clarify the influence relation between and weights of the evaluation criteria, and the evaluation criteria are discussed with regard to three real cases in combination with performance evaluation and improvement based on VIKOR; Section 5 discusses the contributions and limitations of this research and suggests future research directions.

## 2. Establishment of an Evaluation Framework

The WHO defines healthy aging as: developing and maintaining the physical functions of the health of the aged, where physical functions depend on one's endogenous capacity (i.e., the combination of physical and mental abilities) and living environment (including physical, social, and policy environments) and interactions [1]. Based on this, scholars have analyzed the impact mechanism of urban public open spaces on the health of the aged, and have generally summarized several influential factors of open space within three dimensions, namely, outdoor activities, biological affinity, and social support and community place creation [14]. Therefore, in this work, the factors affecting the health and well-being of the aged in green open spaces in old downtown residential communities are summed up through the review of key studies of “outdoor activities,” “connecting with nature,” and “social interaction,” and an evaluation framework is proposed based on this review (as shown in Table 1).

### 2.1. Outdoor Activities

Nature-based outdoor activities play an important role in improving the well-being of the aged [65]. As indicated by many scholars, friendly slow walking paths such as curved ring-type paths can expose the aged to green open spaces for longer periods of time, providing them with more options for outdoor activities, including slow walking, jogging, and brisk walking [24]. Some studies have emphasized that outdoor activities of the aged may be related to safety, walkability, park access, natural and aesthetically pleasing scenery, and recreational facilities [25]. Walkable infrastructures [19], wide barrier-free sidewalks [31], and accessible public transportation are the basic structures required for the aged to develop outdoor activities in green open spaces. A Hong Kong study reported that leisurely walking of local elderly people is positively correlated with connectivity [26]. In addition, the aged prefer to enjoy green open spaces that offer convenient facilities such as services and public toilets [18], as well as well-designed landscapes including comfortable seats for sitting and resting, plants for shade, adequate lighting, and sports related equipment [30]. Physical activity is an important and decisive factor of the health of the aged [34]. Through sports activities, it is possible to extend the time the aged stay in a green open space, further enhancing health and well-being [35]. During the outbreak of COVID-19, appropriate control measures should be taken in response to the fast dissemination and high incidence rate [39]. Studies have shown that the aged can obtain effective and full service information relevant to the surrounding environment from readable signage systems such as air quality monitors, landmarks, or hazard indicators in green open spaces [25]. Meanwhile, some artificial measures such as outdoor ventilators and purifiers can be set up to create a healthy environment. Moreover, separating water supply systems from sewage systems can prevent and delay infection [37]. As confirmed by Thompson and Aspinall [66], outdoor leisure activities can bring benefits to the aged, including physiological benefits for maintaining and enhancing their physical health. All the benefits of nature-based outdoor activities have significant impacts on the well-being of the aged [30].

### 2.2. Touching the Nature (Biological Affinity)

Thompson [66] and Jessica Finlay et al. [44] indicated that connecting with nature is essential for physical and mental health. As jointly shown in several studies, incorporating appropriate features of natural water [37], such as lakes, ponds, and fountains, superior greening vegetation [44], flowers and plants with fragrant smells [42], beautiful outdoor landscapes [30] ,such as small artworks, outdoor furniture, and greening into green open space, can provide the aged with sufficient auditory relief, olfactory treatment, and human immune functions and further improve their active emotions [43]. According to the human-oriented principle, the health and well-being of the aged can be promoted by incorporating biological affinity into green open spaces and giving the aged a sensory experience through interaction with nature [42]. Ratcliffe et al. [46] and Alvarsson et al. [45] stated that pleasant natural sounds such as the sounds of water and wind and chirping of bird songs promote auditory stimulation of the aged; by gardening and planting, the aged not only obtain fruits and vegetables but also experience nature in man-made environments and obtain tactile feedback from real plant life [49]. Douglas et al. [18] mentioned that incorporating leisure and interactive landscapes such as operable landscape installations into the design of green open spaces provides opportunities for the aged to interact with each other, relieves pressure, and produces more active feelings. According to the concept of biological affinity, connecting with nature plays a fundamental role in maintaining the physical and mental health of humans, showing the inner link between humans and nature [67]. In addition, for the aged with weak hearing, no noise is one of the most important factors to ensure smooth communication. Noise increase has a negative impact on communication frequency of the aged, and greatly reduces their expectations from green open space [26].

### 2.3. Social Interaction (Social Support and Community Place Creation)

It has been determined that social interaction, social environment, and social relation (especially in social environments of neighborhood parks) are significantly related with the health of the aged [68]. They are potentially crucial mechanisms for public open space to intervene in the relationship between the environment and the social health of the aged [14]. One of the functions of green open space is to create an outdoor environment for the aged to relax and socialize, and improve their health, well-being, and quality of life [58]. As emphasized in some studies, if the aged can live in more age-appropriate communities with multifunctional open spaces supporting interaction, it is helpful to promote the positive social interaction of the aged [9], reduce depression and anxiety, and improve their quality of life [30]. Studies have shown that in planning, attention should be paid to preserving the elements and cultural heritage of the original community, which is conducive to increasing the sense of belonging and identity [19]. Wong and Yu [64] found that the aged tend to sit down when engaging in social activities. Therefore, providing enough friendly seats and multifunctional spaces and other built-in environmental functions can enable the aged to improve their well-being and sense of self-control through contact and conversation, allowing them more opportunities to develop their social lives, and further establishing strong social connection [25]. Chen [30] pointed out that different levels of social interaction can be achieved through various activities in public open spaces such as dancing, walking, and exercising. Social activities on streets and in community squares are one of the main reasons for the aged to enter public open spaces as they need space to walk and do other sports activities with friends living nearby. Considering that public square space should be available to do interactive activities, it is necessary to make appropriate planning [30]. Social activities of the aged also include keeping pets, which can help release stress in life, reduce depression, and bring a sense of security and love [61]. In particular, walking with pets in public open spaces can help enhance the physical and mental health and social interaction of the aged [69]. Hence, consideration can be taken to set up space for pet activity when designing green open spaces. At the same time, the aged like to visit strongly inclusive places (such as parks or neighborhood open spaces) to watch children playing [59]. Thus, planners should provide the aged with resting areas and wide visibility of children's facilities. This can play a positive role in encouraging the aged to visit parks and such planning allows children and their grandparents to feel safe and enjoy the nature-based entertainment.

## 3. FDANP-mV Model

**Figure 1:**
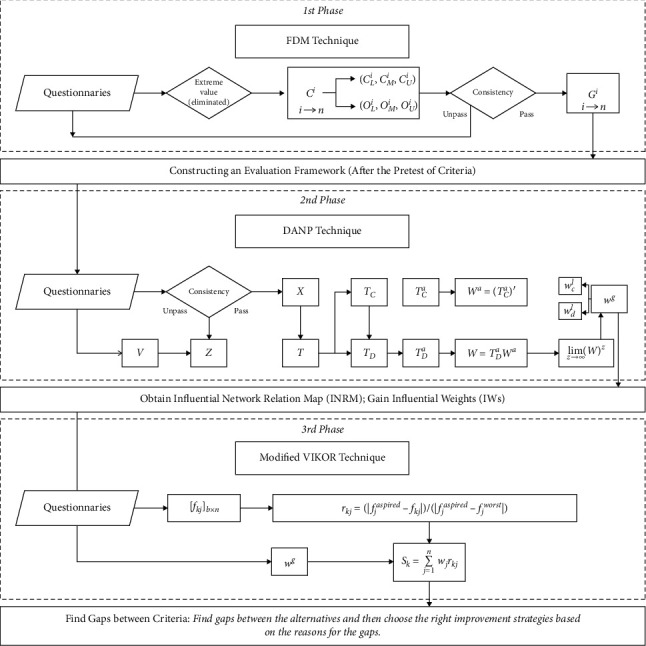
Modeling procedure of FDANP-mV.

The idea of this research is shown in Figure 1. This figure presents the research methods and technical routes applied in different stages of the research and the corresponding research problems. Firstly, an evaluation framework is initially established through a literature review; then, pretest is made using the Fuzzy Delphi Method (FDM) to judge the accuracy of the structure of the framework and the effectiveness of each evaluation criterion; further, the final evaluation framework is constructed. Subsequently, the DANP technique is applied to clarify the influence of relations between various evaluation criteria based on expert knowledge, draw an Influential Network Relation Map (INRM), and allocate the Influential Weights (IWs) of the criteria. In the phase of real case evaluation and analysis, performance evaluation and analyses are made of three old communities in central Guangzhou. Based on the performance evaluation questionnaire, a modified VIKOR technique is applied in this study to determine the gap value between each of the three cases and the total performance value. Above all, analysis results of the research phases are taken as the basis to explore a sustainable and systematic design strategy for improving public open spaces in old downtown neighborhood communities for the purpose of enhancing the health and well-being of the aged.

### 3.1. Fuzzy Delphi Method

Proposed by Dalkey and Helmer [70] in 1960, the Delphi method is a procedural method for systematically expressing the opinions of expert groups. The fuzzy set was incorporated in the Delphi method by Murray, Pipino, and Gigch [71] for the first time. Ishikawa et al. [72] integrated the opinions of experts into fuzzy numbers based on the concepts of cumulative frequency distribution and fuzzy integrals, which is called the Fuzzy Delphi Method (FDM). This method can be used as a tool for screening evaluation criteria. Compared with traditional Delphi method, FDM has the following advantages: (1) the frequency of investigation is reduced; (2) it can completely express the opinions of experts; (3) expert knowledge will be more in line with rationality and practical needs by applying a fuzzy set; and (4) it is more economical in terms of time and cost.

Generally, FDM can be used to carry out the following three main steps: (1) establish a set of evaluation factors that affect decision-making; (2) collect opinions of experts or decision-making groups; and (3) calculate the evaluation value in the FDM method. Zheng Cangbin [73] integrated expert opinions on the basis of a “double-triangular fuzzy number,” and the “grey zone test method” is more effective for testing whether expert opinions are consistently convergent (that is, reaching a consensus). Therefore, in this study, FDM was used to screen out important evaluation criteria for public open spaces in neighborhood communities to improve the health and well-being of the aged, and then to construct an evaluation framework to lay a foundation for subsequent evaluation and analysis. The application and steps of the test method are described in detail as follows:

**Step 1 .**

Count the “conservative value ***C***_i_,” the “optimistic value ***O***_i_,” and the “single value ***A***_i._”

Count the interval value given by the expert for each evaluation criterion in all expert questionnaires. The “minimum value” among the interval values represents the “conservative value ***C***_i_” given by the expert to the quantified score of the evaluation criterion, while the “maximum value” among the interval values represents the “optimistic value ***O***_i_” given by the expert to the quantified score of the evaluation criterion. The single value refers to the “subjective cognitive value” given by the expert to the quantified score of the evaluation criterion.

**Step 2 .**

Take out extreme values that fall outside the “double standard deviation.”

Count the minimum value, maximum value, and the single values given by the experts for each evaluation criterion, and take out the extreme values that fall outside the “double standard deviation.” Then, calculate the remaining minimum value, geometric mean, and maximum value in “conservative value ***C***_i_,” as well as their counterparts in “optimistic value ***O***,” which are not taken out.

**Step 3 .**

Check whether the expert opinions are in agreement with each other based on the “grey zone verification method.”

Establish the triangular fuzzy number (*C*_*L*_^*i*^, *C*_*M*_^*i*^, *C*_*U*_^*i*^) of conservative value ***C***_i_” and that of “optimistic value ***O***_i_” (*O*_*L*_^*i*^, *O*_*M*_^*i*^, *O*_*U*_^*i*^) calculated in Step Two. As per the calculation result shown in Table 2, the interval between the value of *C*_*U*_^*i*^ and that of *O*_*L*_^*i*^ refers to the verification value of ***M***_i_—***Z***_i_ for the grey zone. Where *M*_*i*_=(*O*_*M*_^*i*^ − *C*_*M*_^*i*^), *Z*_*i*_=(*C*_*U*_^*i*^ − *O*_*L*_^*i*^). In the case that the value of *C*_*U*_^*i*^ is larger than *O*_*L*_^*i*^, and ***M***_i_—***Z***_i_ is bigger than 0, it indicates that the expert opinions tend to be identical, and convergence is reached for the evaluation criterion; when ***M***_i_—***Z***_i_ is negative, it means that the expert opinions are not in agreement with each other, and no convergence is reached for the evaluation criterion. At this point, the second questionnaire can be used for the evaluation criterion for which convergence has not yet been reached.

**Step 4 .**

Calculate the consensus value of the expert ***G***_i_, and identify the reasonable threshold.

The intersection node of the two lines of the *C*_*U*_^*i*^ value and *O*_*L*_^*i*^ value refers to the consensus value of the expert *G* ^*i*^, which presents an important basis for the identification of the threshold. In the meantime, the higher the value of *G* ^*i*^, the higher the consensus value of the expert, or, in other words, the higher the importance of the evaluation criterion. It can be seen from Table 2 that in this study, the threshold is set at 6.667, referring to the arithmetic mean of *G* ^*i*^, and is based on the semantic scale of a Likert questionnaire. Therefore, each criterion for which *G* ^*i*^ is lower than the threshold of 6.667 is taken out.

### 3.2. Research Procedures of the DANP Technique

In 1996, Thomas L. Saaty established the Analytic Network Process (ANP) with attribute dependency and feedback relation based on the issue of improving the Analytic Hierarchy Process, AHP, thereby bringing the weight of attributes closer to the real-world condition. However, in ANP, it is assumed that the attribute weight value of each facet is equal. Though this simplifies calculation, the fact that the importance of the attribute is not equal to that in the real world is neglected. As a result, in 2008, based on the Markov Chain Process (the degree of importance is characterized by Transferability), Gwo-Hshiung Tzeng stressed the degree of importance for the calculation of attributes through the ANP mode according to the total influence relation matrix ***T*** established by DEMATEL as the influence weight. Through the empirical analysis, Tzeng showed that this method better conforms with the conditions of real society.

**Step 5 .**

Create direct relation matrix ***Y***.

The experts transform the experience of real society into the degree value of the interaction of the attributes within the model structure based on five point Likert scales (0 for no influence, 1 for very low influence, 2 for low influence, 3 for high influence, and 4 for very high influence) and establish the receptance matrix ***Y*** for the direct relation matrix with nonnegative value of *n* × *n*, where *y*_*ij*_ represents the degree of the impact of the i-th criterion on the j-th criterion.

**Step 6 .**

Create initial direct relation matrix ***Z***.

Use equations (1) and (3) to integrate the direct relation matrix of ***Y*** for *p* experts, thereby obtaining initial direct influence relation matrix ***Z*** representing the experience of *p* experts, where *p* refers to the total number of experts and *y*_*ij*_ represents the degree of the impact of the *i*-th criterion on the *j*-th criterion.

(1) $$\begin{array}{c} Z_{ij}=\frac{1}{p}\sum_{k=1}^{p}y_{ij}^{k}, \end{array}$$

(2) $$\begin{array}{c} Z=\left[\begin{array}{ccccc} z_{11} & \cdots & z_{1j} & \cdots & z_{1n}\\ \vdots & & \vdots & & \vdots \\ z_{i1} & \cdots & z_{ij} & \cdots & z_{in}\\ \vdots & & \vdots & & \vdots \\ z_{n1} & \cdots & z_{nj} & \cdots & z_{nn} \end{array}\right]. \end{array}$$

**Step 7 .**

Examine the consensus.

The value of consensus can be estimated by equation (3), which represents the level of expert consensus. The threshold of the average gap ratio is 5%, and a value less than 5% implies a confidence level above 95%, which also represents a stable system. Conversely, if an unstable system is obtained, the first phase should be implemented again to verify whether data collection is correct and whether the number of experts is sufficient.

(3) $$\begin{array}{c} \text{average gap}-\text{ratio in consensus }\left(\%\right)=\frac{1}{n\left(n-1\right)}\sum_{i=1}^{n}\sum_{j=1}^{n}\left(\frac{\left|z_{ij}^{p}-z_{ij}^{p-1}\right|}{z_{ij}^{p}}\right)\times 100\%. \end{array}$$

**Step 8 .**

Normalize direct influence matrix ***X***.

The initialization of direct influence relation matrix Z can build the boundary of its influence relation matrix through equations (4) and (5). All the influence values of its influence relation matrix are within the range from 0–1; after that, the standardized direct relation matrix ***X*** has a minimum row-column total value of 0, and a maximum value of 1.

(4) $$\begin{array}{c} X=m\cdot Z, \end{array}$$

(5) $$\begin{array}{c} m=\min\left\{\frac{1}{\max_{1\;\leq i\leq n}\sum_{j=1}^{n}z_{ij}},\frac{1}{\max_{1\;\leq j\leq n}\sum_{i=1}^{n}z_{ij}}\right\}. \end{array}$$

**Step 9 .**

Obtain total influence relation matrix ***T***.

The standardized direct relation matrix ***X*** is able to calculate the value of multiple influences and the indirect influence of the criterion, i.e., the total influence value of the criterion, through equation (6). Therefore, when **o**⟶*∞*, that is, **N** ^*o*^=[0]_*n*×*n*_, the total influence relation matrix ***T*** is obtained, where ***I*** refers to the unit matrix.

(6) $$\begin{array}{c} T=X+X^{2}+X^{3}+\cdots +X^{u},whereo⟶\infty ,X^{o}={\left[0\right]}_{n\times n},\\ =X\left(I—X^{k-1}\right){\left(I—X\right)}^{-1},\\ =X{\left(I—X\right)}^{-1}. \end{array}$$

**Step 10 .**

Establish the network relationship map (NRM).

At last, the total value of attributed influence degree *r*_*i*_ is obtained by summing up the attribute column of the obtained total influence relation matrix ***T***. The summing up of the row refers to the total value of the degree of the influenced attribute *d*_*i*_, while *r*_*i*_ + *d*_*i*_ refers to the correlation degree of the attribute, which is called the prominence; *r*_*i*_ -*d*_*i*_ denotes the degree of significance for the influence nature of the attribute, which is called the relation. In addition, a method for quantifying the system structure relation is available for the efficient simplification of the degree of complexity of inter-criterion correlation, thereby making the overall perspective of the entire evaluation system easy to understand and utilize.

**Step 11 .**

Establish the unweighted supermatrix **W** ^*a*^.

Normalize the total influence relation matrix **T**_*C*_ by the dimensions (called “clusters”) as shown in the following equation:
(7)

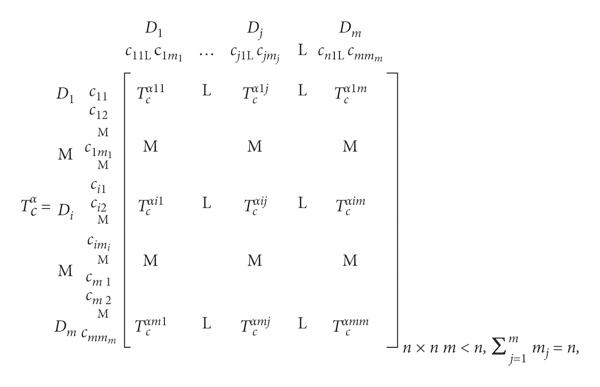

where **T**_*C*_^*α*^ denotes the normalizing total influence relation matrix of criteria by dimensions, and *T*_*c*_^*α*14^ is derived from equations (8) and (9). Similarly, *T*_*c*_^*αmm*^ can be obtained as follows:

(8) $$\begin{array}{c} t_{i}^{14}=\sum_{j=1}^{m_{4}}t_{ij}^{14},\;i=1,2,\ldots ,m_{1}, \end{array}$$

 
(9)

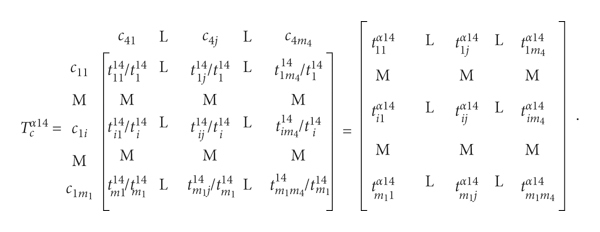

First, a normalization calculation was performed on the total criterion influence relationships according to different dimensions. Since the direction of pairwise comparison between the criteria is opposite to the ANP concept, the total-influenced relationship matrix needs to be transposed. The total-influenced relationship matrix is obtained from the DEMATEL, and each column is summed up for normalization. After normalization, an unweighted super matrix is established.

**Step 12 .**

Establish the weighted supermatrix **W**_*w*_.

The weighted super matrix **W**_*w*_ can be obtained by multiplying the criterion of **W** ^*a*^ in the unweighted supper matrix and the facet of **T**_*D*_^*α*^, as shown in equation (10).
(10)

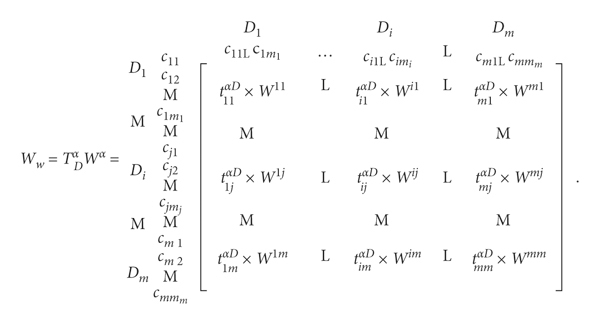

**Step 13 .**

Calculate the limiting supermatrix **W** ^g^.

Convergence and stability can eventually be achieved through self-multiplication of the weighted supper matrix **W**_*w*_, or, in other words, by obtaining the maximized super matrix **W** ^g^ where **W**_*w*_ refers to the weighted super matrix and *l* represents the number of self-multiplications.

### 3.3. Research Procedures of the Modified VIKOR Technique

VIKOR (VlseKriterijumska Optimizacija I Kompromisno Resenje) is a multi-criteria, decision-making method that was proposed by Professor Serafim Opricovic and Professor Gwo-Hshiung Tzeng in 1998. VIKOR is one of the optimal compromise solutions of multi-criteria decision-making methods, the basic idea of which is to first define the positive-ideal solution (optimal solution) and the negative-ideal solution (the worst solution).

**Step 14 .**

The modified approach for replacement by the aspiration level and the worst value is as follows.

The aspiration level: *f* ^aspired^=(*f*_1_^aspired^,…, *f*_*j*_^aspired^,…, *f*_*n*_^aspired^), where *f*_*j*_^aspired^ is an aspiration level, which is also called the best value.

The worst values: **f** ^worst^=(*f*_1_^worst^,…, *f*_*j*_^worst^,…, *f*_*n*_^worst^), where *f*_*j*_^worst^ is a worst value.

In this study, performance scores ranging from 1 to 5 (very bad ⟵ 1, 2,…, 5 ⟶ very good) are used with natural language in the linguistic/semantic questionnaire. Thus, the aspiration level takes the highest score of 5 and the worst value takes the value of 1. Hence, *f*_*j*_^aspired^=5 is defined as the aspiration level and *f*_*j*_^worst^=1 is defined as the worst value.

**Step 15 .**

Determine the mean group utility for the gap and then establish the priority improvement strategy. These values can be calculated using the following equation:

(11) $$\begin{array}{c} s_{k}=\sum_{j=1}^{n}w_{j}r_{kj}=\frac{\sum_{j=1}^{n}w_{j}\left(\left|f_{j}^{\text{aspired}}-f_{kj}\right|\right)}{\left(\left|f_{j}^{\text{aspired}}-f_{j}^{\text{worst}}\right|\right)}, \end{array}$$

where *s*_*k*_ is defined as the normalized ratio (%) of distance to the aspiration level, which implies the synthesized gap of the criteria. In this case, *w*_*j*_ indicates the IWs for the criteria obtained from DANP.

## 4. Results and Discussion

### 4.1. Using FDM to Effectively Screen Criteria

In this research, the design of the fuzzy Delphi questionnaire was based on related literature, and the content was oriented to the design strategies to improve the health and well-being of the aged in green open spaces old downtown residential communities. Researchers summarized the relevant influential elements acquired by close reading (of the interview transcript verbatim) and considering the multiple meanings inherent in the text. In the analysis process, the meanings were described in text (such as correlation, chain, and implication) initially through continuous analysis and comparison on the modification and improvement of each influential element. Furthermore, the elements with similar meanings were combined in categories (i.e., outdoor activities, touching the nature, and social interaction). Through inductive analysis of related materials, 23 influential elements were extracted, and each design element was provided with supplementary instruction as per the coding records, as shown in Table 1.

The 23 design influential elements in Table 1 were further screened and analyzed to make clear their effectiveness in serving as evaluation criteria. Moreover, the influential elements were screened using the FDM method; experts from relevant fields were invited to fill out the fuzzy Delphi questionnaire for experts according to a scale of 1–10 as per the importance of each influential element, wherein the experts had no less than 5 years of working experience and engaged teaching related to environment art design, scientific research, and architectural landscape design. Finally, a total of 38 questionnaires were distributed and 30 valid questionnaires were collected. In this research, FDM was used to integrate expert opinions based on “double-triangular fuzzy numbers,” and the “grey zone test” was taken as an effective way to test the consistency of the results recognized by experts. Based on the semantic scale of a Likert questionnaire, 6.667 was determined as the threshold value of consistency in this research. As a result, among the 23 influential elements, 3 elements (**C**_3_; **C**_14_; **C**_21_) scored in the range of 6.5–7, 6 elements (**C**_6_; **C**_9_; **C**_12_; **C**_13_; **C**_18_; **C**_20_) scored in the range of 7.1–7.5, 7 elements (**C**_4_; **C**_5_; **C**_7_; **C**_8_; **C**_10_; **C**_11_; **C**_22_) scored in the range of 7.6–8, and 7 elements (**C**_1_; **C**_2_; **C**_15_; **C**_16_; **C**_17_; **C**_19_; **C**_23_) scored above 8. The second round of expert interviews only addressed the three influential elements that scored in the range of 6.5–7. On the basis of the interview, the fuzzy Delphi questionnaire was distributed again to confirm the importance of the three elements. The analysis results show that the consistence values of **C**_14_ and **C**_21_ fail to pass the threshold so that they are excluded from the effective influential criteria. The remaining 21 influential elements can be used as important evaluation criteria for reconstruction of green open spaces in old downtown residential communities. The statistical results are shown in Table 2. The expert consistence values of each criterion are listed in the rightmost column.

### 4.2. Using DANP to Clarify the Influence Relation between and Weights of Evaluation Criteria

**Figure 2:**
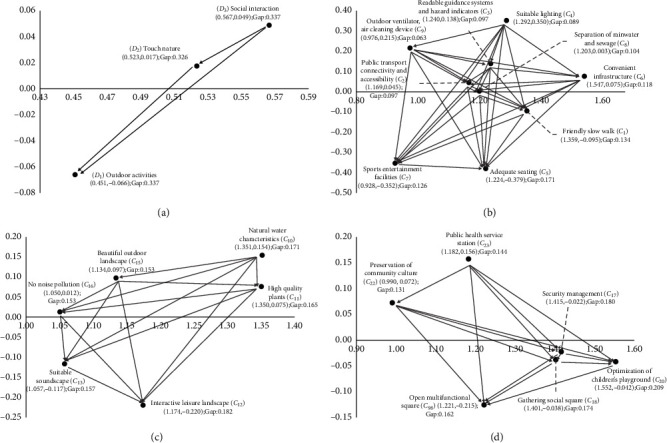
The INRM (influential network relation map) of the total influence relationships.

Based on Fuzzy Delphi, the effectiveness of criteria was screened and ***C***_14_ and ***C***_21_ were evaluated so that they could be excluded from the effective criteria. In this section, the findings show how the DANP-V model can clarify the influence relationships of the screened criteria and allocate weights. This survey distributed 32 DEMATEL questionnaires. Some of these questionnaires were collected through expert group meetings and were based on the results of expert discussions. The consistency reached more than 96% by the end of the calculation. Therefore, 27 valid expert questionnaires were selected. The INRM in Figure 2 clearly expresses the influence network relationship structure of the interaction in the improved model. INRM indicates that the impact priority of these three dimensions can be sorted in the following order: Social Interaction (***D***_3_) > Connect with Nature (***D***_2_) > Outdoor Activities (***D***_1_), and Outdoor Activities (***D***_1_) were influenced by Social Interaction (***D***_3_) and Connect with Nature (***D***_2_), whereas Connect with Nature (***D***_2_) was influenced by Social Interaction (***D***_3_). This result shows that when considering improving the dimensions of Outdoor Activities (***D***_1_), the Social Interaction (***D***_3_) and Connect with Nature (***D***_2_) dimensions should be emphasized. In addition, in order to improve the Connect with Nature (***D***_2_) dimension of the green space, improving the standards of the Social Interaction (***D***_3_) dimension should be highlighted. Similarly, from the perspective of INRM, the most influential criterion for Outdoor Activities (***D***_1_) is Suitable lighting (***C***_4_), and the order of influence of the remaining criteria is ***C***_9_, ***C***_3_, ***C***_6_, ***C***_2_, ***C***_8_, ***C***_1_, ***C***_7_, and ***C***_5_. Natural water characteristics (***C***_10_) was the most powerful criterion in the Connect with Nature (***D***_2_) dimension, and the order of influence of the remaining criteria is ***C***_15_, ***C***_11_, ***C***_16_, ***C***_13_, and ***C***_12_. Public health service station (***C***_23_) was the most powerful criterion in the Social Interaction (***D***_3_) dimension, and the order of influence of the remaining criteria is ***C***_22_, ***C***_17_, ***C***_18_, ***C***_20_, and ***C***_19_.

### 4.3. Empirical Cases

This section introduces the green open space cases of three communities among old downtown residential communities in a high-density city represented by Guangzhou, as well as the data collection and analysis process. In addition, sustainable and systematic improvement strategies for each real case are discussed on the basis of INRM and current performance evaluation results, taking INRM and IWs as the standards and expecting to enhance the health and well-being of the aged.

#### 4.3.1. Old Nanhai County Community on Liurong Street

**Figure 3:**
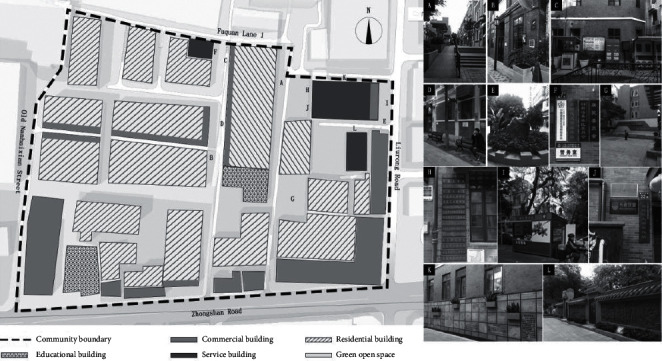
Plan of the Old Nanhai County Community on Liurong street.

Under the jurisdiction of the Office of Liurong Street, Yuexiu District, Guangzhou City, Old Nanhai County Community has a total land area of about 4.28 hectares, with about 1,840 resident households and 5,155 residents. The aged residents account for about 38.79% of the population. The plan is shown in Figure 3. In the dimension of outdoor activities, most of the slow walking paths in the community are provided next to buildings (Part A of Figure 3). Having a long history, the community borders Qing General's Office site on Liurong Road in the east, Zhongshan Road in the south, Old Nanhai County Street in the west, and Liurong Temple on Fuquan Lane 1 in the north. The surrounding public transportation is well connected and accessible. After a micro reconstruction, the community is now equipped with relatively complete lighting and guidance systems (Part B of Figure 3) as well as infrastructures such as garbage-sorting stations and public toilets (Part C and Part D of Figure 3). A community square is provided with a space for fitness activity. The sewage system is well maintained. However, under the epidemic, there is lack of related outdoor ventilation machines and air cleaning devices. In the dimension of connecting with nature, the reconstruction did not take full consideration of creating natural water landscapes, soundscapes, plant arrangements, and the like due to the orientation toward cultural inheritance (Part E of Figure 3). However, there are complete interactive landscapes and outdoor landscapes. No obvious source of noise can be found in the community. In the dimension of social interaction, the community is a cultural propaganda demonstration community, with complete safety management facilities (such as monitoring systems) (Part F of Figure 3) as well as an activity square for residents to relax and socialize, multifunctional open spaces, and public health service stations (Part G, Part H, Part I, and Part J of Figure 3). Unfortunately, no spaces for children to play were found in the field investigation. The cultural heritage preservation design is worthy of reference for reconstruction of other communities (Part K and Part L of Figure 3).

#### 4.3.2. Panqiu Community on Jinhua Street

**Figure 4:**
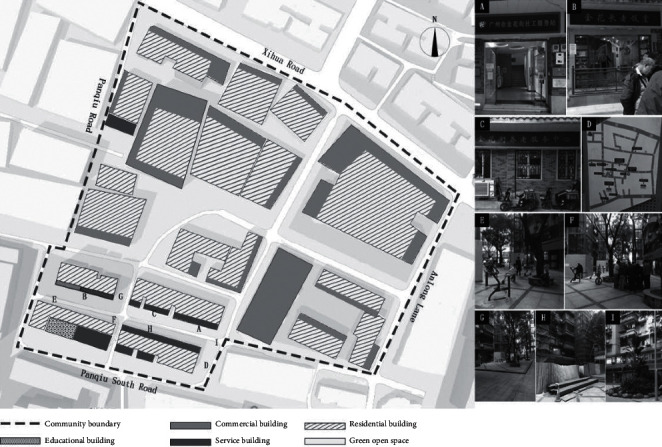
Plan of the Panqiu Community on Jinhua street.

Located on Jinhua Street, Liwan District, Guangzhou City, Guangdong Province and spanning from Anlongli, Xihua Road in the east and Ludi Street, North Guangfu Road in the south, to Panqiu Street, Xihua Road in the west and Xihua Road in the north, the Panqiu Community has a total area of about 0.089 square kilometers, with 1,478 permanent resident households, and 4,418 residents in total. The plan is shown in Figure 4. Jinhua Street Nursing Home and Jinhua Cultural Plaza falls within the area and most of the people active in the community are aged. Hence, well-being facilities and public infrastructures can be guaranteed (Part A, Part B, and Part C of Figure 4). In the community, there are complete tourism, culture, health care, education, and shopping facilities, and a good guidance system, instructions, traffic connection, and accessibility (Part D of Figure 4). A cultural square is provided with sports and fitness facilities and recreational areas (Part E and Part F of Figure 4). However, there is no children's playground. In the old downtown area of Guangzhou, micro-reconstruction activities have been carried out in recent years. Subsequently, the underground pipeline facilities of the community have been repaired, the existing lighting system is relatively complete, and the roads (including slow walking path) are well maintained (Part G of Figure 4). But with the outbreak, outdoor ventilation machines and air cleaning devices are still lacking. As a gathering area for the aged, the community was mainly reconstructed along humanity orientation lines. Safety management facilities (such as monitoring systems), public health service stations, and other facilities are in relatively good condition. Elevators are being added to old buildings in the community. So, noise mainly comes from the construction (Part H of Figure 4). However, the design considerations of plant arrangement, natural water landscapes, soundscapes, and other aspects remain to be improved (Part I of Figure 4). It was this community's focus on public infrastructure for the aged that provided the idea for conducting this research.

#### 4.3.3. Sanfu Community on Jinhua Street

**Figure 5:**
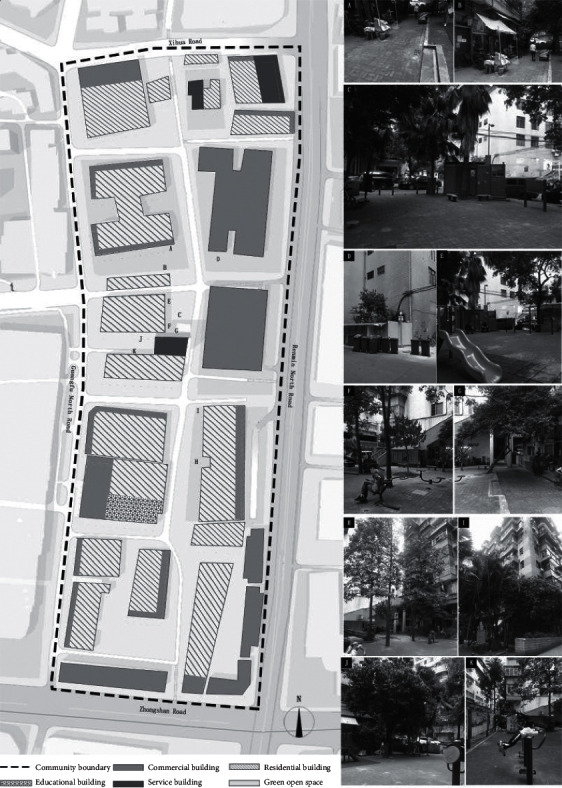
Plan of Sanfu community on Jinhua street.

Located east of Jinhua Street, Liwan District, Guangzhou and bordering North Renmin Road in the east, North Guangfu Road in the west, 7th Zhongshan Road in the south, and Xihua Road in the north (the plan is shown in Figure 5), the Sanfu Community has a total area of about 0.01 square kilometers, with 1,520 resident households, 4,626 permanent residents, and 1097 immigrant residents. A total of 303 households are not registered in the community. The total population has reached up to 5,723 persons. The neighborhood committee currently involves 28 relief households and 14 low-income households. In addition, 72 persons with disabilities are registered. The current situation of public open spaces in the community is as follows. First of all, in the dimension of outdoor activities, most of the walking paths in the community are provided around residential buildings, and are shared by people and vehicles. However, many of these places do not provide barrier-free facilities (Part A and Part B of Figure 5). Public transportation around the community is well connected and accessible, and there are multiple bus lines and subways passing through. Rest seats and lighting existing in the community are old and somewhat damaged. No specific landscape lights are provided in the public open space (Part C of Figure 5). But in terms of guidance system (15-minute living circle in the community), there is relatively complete infrastructure such as a garbage sorting station (Part D of Figure 5). Public open spaces in the community are equipped with fitness equipment donated by the society and government (Part E and Part F of Figure 5). Under the outbreak, no outdoor ventilation machines and air cleaning devices have been installed; but measures such as regular manual spraying for disinfection are taken. In the dimension of connecting with nature, due to the impact of limited land for use, there is no large lawn area but merely a few small trees scattered and irregularly distributed along the street and in public open space. Plant arrangement, natural water landscapes, soundscapes, and the like are not reflected in the public open spaces of the community (Part G, Part H, Part I, and Part J of Figure 5). No obvious noise source is found. In the dimension of social interaction, safety management facilities (such as monitoring systems) are relatively complete. A paved square is the main form of public open space in the community and can be used to develop various activities and leisure and social interaction among residents (Part C of Figure 5). No space for children to play was found. However, field investigation found that most children play in the fitness activity area (Part K of Figure 5). The old city wall and the characteristic buildings of Xi Guan make up the landscape and cultural features of the community, but they are not embodied in the public open space.

#### 4.3.4. Using VIKOR to Make Performance Evaluation and Improvement

On the basis of INRM and IWs discovered by DANP, we can ensure performance and improvement by using the VIKOR technique and further formulate sustainable and systematic strategies for improving green open space in real cases so as to promote healthy aging.  Table 3 lists the performance and gap values of the dimensions/criteria in the three real cases. We assume that when the gap values are improved, the performance of each dimension/criterion will increase accordingly, and the overall case will tend to reach the desired level. Traditional discussions on evaluation and improvement often focus on the most significant practical problems (the largest gap), and the priority of improvement is also determined on this basis. However, the DANP-V model uses a systematic method to clarify the influence relation between the criteria and discover the influential source of the practical problems.

In the Old Nanhai County Community, the gap values of the three dimensions are ranked from large to small as follows: connecting with nature (**D**_2_), outdoor activities (**D**_1_), and social interaction (**D**_3_). As shown in Table 3, natural water feature (**C**_10_) has the largest gap value (0.5545) in **D**_2_ and also has strong IW. In comparison, this community currently has good, friendly slow walking paths, and has also obtained high satisfaction in terms of safety management and cultural preservation of the community. More importantly, INRM shows that **C**_10_ is the most dominant criterion in the specific dimension. Therefore, for improvement of the case, decision-makers should allocate more resources to natural water features (**C**_10_) to enhance the performance of the case in other criteria. On the other hand, the influence of **D**_3_ on **D**_2_ reminds decision-makers that they should pay more attention to some of the problems presented in the dimension of social interaction. For example, Table 3 shows that the community has poor performance in **C**_18_ and **C**_20_. This implies that decision-makers can think about how to meet people's demands for social interaction and children's entertainment based on the hydrophilicity of people. This could not only improve dimension **D**_3_ but also drive improvement of **D**_2_.

In the Sanfu Community, the gap values of the three dimensions are ranked from large to small as follows: connecting with nature (**D**_2_), outdoor activities (**D**_1_) and social interaction (**D**_3_). Except for **C**_10_, the community performs poorly in superior vegetation (**C**_11_) under the second dimension, and performs the worst in outdoor ventilation and air cleaning (**C**_9_) under **D**_1_. However, currently, the community has good traffic conditions and accessibility, and good performance in safety management and noise control. Figure 2 shows that in addition to incorporating a natural water element into the community, decision-makers should also pay attention to the aesthetics of the physical environment, including selection of plant species in the field, consideration of configuration and decoration, color matching, form arrangements, etc. Similarly, decision-makers should also start promoting social communication and guaranteeing healthy rights and interests of the residents, providing more convenient service facilities and open squares or pocket parks with good atmospheric conditions. Ventilation conditions of the structures and associated facilities should also be taken into consideration in the planning and design.

As can be seen from the performance results, the Panqiu Community also has the worst performance in **D**_2_ (0.5602), and slightly better performance in **D**_3_ (0.3476) than in **D**_1_. In addition to natural water features (**C**_10_), the community also performs poorly in noise pollution conditions under **D**_2_. At the same time, the community currently does not have high-quality plant arrangement and cannot form an interactive landscape for residents to relax. In **D**_1_, air quality, convenient basic service facilities, and treatment of rainwater and sewage in the community cannot be well guaranteed to the residents. However, the community currently has good conditions and accessibility and can provide the residents with good safety management and health services. Through field investigation, it was revealed that the current noise pollution in the community is due to the government-supported project for installing elevators in old residential buildings. The ongoing reconstruction project does not involve the overall consideration of high-quality plants, and it is even foreseeable that the project will occupy green area and public activity space.

The performance results show that among the three real cases, Sanfu Community is least suited to improving health and well-being construction for the aged, while the current development of better community is Old Nanhai County. However, the three real cases show high degrees of consistency in the three dimensions of performance; the notable problems are concentrated in **D**_2_. Combining practical experience, it can be deduced that green open spaces in the old downtown residential communities of Guangzhou generally lack resource input and planning and design considerations in the aspect of connecting with nature. However, Douglas et al. [18] and Azadeh Lak et al. [19] have expressed that connecting with nature plays a fundamental role in the physical and mental health of the aged and accomplishes the internal link between humans and nature. Thereby, it is believed that in follow-up planning of old downtown reconstruction, decision-makers should try to improve the opportunities and quality of connecting with nature for the aged in old communities in order to improve their health and well-being. Furthermore, we propose incorporating natural blue and green elements into the design of public living spaces in communities from the perspective of social interaction and care based on old people's preference for natural water environments and ornamental plants and the creation of interactive landscapes. To sum up, a bottom-up observation of the behavioral habits, perception needs, and psychological preferences of the aged in old communities is the key foundation for creating good outdoor activities and natural landscapes in green open spaces of communities. Especially after the outbreak of COVID-19, decision-makers should strengthen safety guarantees for social interaction of the aged in communities, and update the previous practices and contents of social activities on the basis of meeting the needs of the aged to practically create healthy, safe, and well-used outdoor activity spaces and natural landscapes with high ornamental value and interactivity.

## 5. Conclusions

This study aims to provide theoretical research and practical improvement strategies for improving the health and well-being of the aged in old downtown residential communities. First of all, 23 elements of green open spaces that can affect the health of the aged were extracted from previous literature and divided into three dimensions, namely, outdoor activities (***D***_1_), connecting with nature (***D***_2_), and social interaction (***D***_3_). Second, applicability and effectiveness of the 23 criteria were judged on the basis of expert knowledge. On this basis, an evaluation framework for evaluating green open spaces in old downtown residential communities was constructed. Combined with public health and urban design, the evaluation framework was further used to view the status quo of community environments and provide decision-making strategies for improving the health and well-being of the aged. Third, the influence relation and priority between the dimensions and criteria of the evaluation framework were clarified. In an effort to loosen the independence between the criteria, an Influential Network Relation Map (INRM) was drawn, which made it possible to not only recognize the current dominant problems by situation evaluation analysis but also to identify the influential sources of the problems by clarifying the dominant influential relation. On this basis, improvement strategy was formulated, making sure to avoid the disadvantages of “taking stopgap measures” in traditional evaluation research and averting waste to the maximum extent possible on the premise of limited resources. Finally, taking Old Nanhai County Community, Sanfu Community, and Panqiu Community as representative real cases, performance evaluation was made to explore the focus issues existing in reality, and discussion of sustainable and systematic improvement strategies applicable to each of the communities was done based on INRM.

The limitations of this research should be acknowledged, namely, based on performance evaluation analysis, the improvement strategies explored in this research are only formulated for the three residential communities in the old downtown area of Guangzhou. Therefore, it is not recommended to apply these improvement strategies in relevant cases in other regions. However, the process of developing improvement strategies and the evaluation framework provided in this research can be used as a reference for investigating improvement priorities of residential communities in many areas. In addition, the modified VIKOR applied in this research is an additive performance evaluation method, while nonadditive situations are often the case in reality. Hence, in follow-up research, nonadditive performance evaluation methods can be taken to get closer to the real situation. On the other hand, if objective data can be collected by investigating a large number of old downtown residential communities, it would be possible to further make clear the core of the evaluation framework constructed in this research by using data exploration techniques such as Rough Set Approach (RSA) and clarifying the decision rule (if-then) in the investigated data based on the research results. Moreover, in combination with expert knowledge, the DEMATEL technique could be used to explore “decision-making path maps” designed for green open spaces in old downtown residential communities to improve the health and well-being of the aged.

## Figures and Tables

**Table 1:** Index factors for pretest.

Dimensions	Criteria	Description	Reference source
Outdoor activities (***D***_1_)	Friendly slow walking (***C***_1_)	Providing continuous circular or curved walking paths for the aged to do more outdoor activities such as slow walking, jogging, and brisk walking; eliminating any form of slope or puddle on the walking path, and constructing walk-friendly infrastructure to get rid of the fear of falling of the aged; providing barrier-free sidewalks with sufficient width to meet the needs of special elderly people.	[19, 24, 25]
	Public transport connectivity and accessibility (***C***_2_)	Ensuring the public traffic routes adjacent to the green open space are clear and well-connected so that it is easy to find and enter the space; and routes should be within suitable walking distance from the space.	[26–28]
	Readable guidance systems and hazard indicators (***C***_3_)	Setting up information exchange boards, air-quality test displays, landmark or hazard indicators to provide the aged with effective and adequate service information about the surrounding environment.	[25, 29, 30]
	Suitable lighting (***C***_4_)	Choosing applicable lamps, setting the brightness and installing the lamps in appropriate positions to ensure suitable brightness transition and uniformity, and clear vision, so as to enhance safety in night activities.	[19, 25]
	Adequate seating (***C***_5_)	Providing comfortable seats with backrests and armrests for the aged to stay and rest and communicate with each other (the seats should be installed as per the overall green open space design, facing wide views; the backs should be shaded by tree shadows. The seats can be small benches with low soft walls or sheltered landscape structures such as gallery frames).	[30–33]
	Convenient infrastructure (***C***_6_)	Providing drinking water sources, public toilets and the like provisions for convenience and environmental cleanliness, which are considered as the most important needs of the aged.	[18,21]
	Sports entertainment facilities (***C***_7_)	Recreational facilities can be used, such as outdoor sports equipment, golf courses, or bicycle lanes. The aged prefer recreational facilities with guidance and emergency braking systems to avoid injury.	[24, 34, 35]
	Separation of rainwater and sewage (***C***_8_)	Historically, constructive measures and standards for preventing and delaying epidemics mainly include urban and rural water supplies and sewage systems (separation of rainwater and sewage).	[36–38]
	Outdoor ventilator/air cleaning device (***C***_9_)	COVID-19 can spread through droplets under windless conditions; hence, it is necessary to take some manual measures to enhance ventilation efficiency, quickly remove air pollutants, and further prevent the spread of the virus.	[39–41]

Touching the nature (***D***_2_)	Features of natural water (***C***_10_)	Appropriate features of natural water such as lakes, ponds, and fountains should be provided, as water elements in nature can lead to higher therapeutic responses and preferences and improve active emotions in the aged.	[19, 33]
	High-quality plants (***C***_11_)	Vegetation planted in suitable positions and suitable greening allocation throughout the four seasons can offer sufficient visual relief effects through adjustment of natural colors and the sun. Planting flowers and plants with fragrant smells is conducive to improving the olfactory and immune functions of the aged. To avoid pollen allergies, it is necessary to choose flower varieties with less pollen.	[42–44]
	Interactive leisure landscape (***C***_12_)	Incorporating opportunities for incidental and leisure interaction with the environment into the design of green open spaces, such as providing operable landscape installations to encourage interaction.	[18]
	Suitable soundscape (***C***_13_)	Providing pleasant natural sounds such as birdsongs (trees suitable for perching birds can be planted) and the sounds of water and wind (waterscapes or microclimates can be created by virtue of altitude difference) to stimulate auditory sense and promote faster physiological recovery.	[45–48]
	Access to fruits and vegetables (***C***_14_)	Making available fruit and vegetable plants though gardening and DIY so that the aged can obtain tactile feedback from real plant life in nature, taste fresh fruits and vegetables, and have more positive feelings.	[49, 50]
	Beautiful outdoor landscape (***C***_15_)	Providing beautiful outdoor landscapes in harmony with the natural environment, such as the combination of greening and street furniture and small artworks to achieve humanistic ecological benefits and increase the interest and artistry of the landscapes.	[19, 51]
	No noise pollution (***C***_16_)	The aged in high-density urban areas prefer quiet green open spaces. No noise is one of the most important factors to ensure smooth communication, especially for the aged with poor hearing. The increase in noise may negatively affect the communication frequency of the aged.	[52–55]

Social Interaction（ ***D***_3_）	Security management (***C***_17_)	Including daily patrols for security, installation of monitoring TV, and maintenance of infrastructures such as changing guardrails along waterfronts and replacing old exercise equipment to avoid crime and vandalism.	[32, 56, 57]
	Gathering social square (***C***_18_)	Forming a large square space for the aged to carry out various entertainment activities, and adding wide spaces for promoting their participation in and supporting community communication.	[[25, 30, 33]
	Open multi-functional spaces (***C***_19_)	The wider the adjacent street is, the stronger the sense of openness is, and the lower the surrounding buildings are, the stronger the sense of openness is. This can help increase the frequency of social interaction.	[40,58]
	Optimization of children's playground (***C***_20_)	The children's play area should have good visibility and a space suitable for the aged to rest. This can drive inter-generational exchanges.	[59]
	Pet activity space (***C***_21_)	Giving full consideration to pet activity space to increase the outdoor activities of the aged and drive the aged to take walks with their pets in the space to enhance their physical and mental health; this has been found to be a way to lower the blood pressure of the aged and help strengthen community exchange and interaction.	[60–62]
	Preservation of community culture (***C***_22_)	The full application of elements with cultural and historical information of the original community to the design of green open spaces can enhance local identity and sense of belonging.	[25, 63, 64]
	Public health service station (***C***_23_)	This kind of station functions to quickly and conveniently deal with the emergency health conditions of the aged while disseminating daily disease prevention knowledge, and facilitates good connectivity and accessibility. Necessary space for emergency shelters should be established to deal with sudden disasters, and obvious route maps should be posted.	[6, 37]

**Table 2:** Fuzzy Delphi statistical analysis results.

Criteria	Conservative Value *C*i		Optimistic Value *O*i		Single Value *A*i		Geometric Mean M			Verification values	Consensus values
	Min	Max	Min	Max	Min	Max	*C*i	*O*i	*A*i	*M*i*—Z*i	*G*i
Friendly slow walking **(*C***_1_)	5	8.5	8	10	6.5	9	6.621	8.811	7.586	1.690	8.151
Public transport connectivity and accessibility (***C***_2_)	6	8.2	8	10	7	9	6.891	9.005	7.869	1.914	8.087
Readable guidance systems and hazard indicators (***C***_3_)	4	8	6	9	5	8	5.525	7.813	6.609	0.288	6.846
Suitable lighting (***C***_4_)	5	9	7.5	10	6	9	6.357	8.644	7.449	0.787	7.953
Adequate seating (***C***_5_)	5	9	7	10	6	10	6.447	8.528	7.335	0.081	7.749
Convenient infrastructure (***C***_6_)	5	8	7	10	6	9	6.149	8.594	7.148	1.445	7.463
Sports entertainment facilities (***C***_7_)	5	9	7	10	6	9	6.578	8.655	7.643	0.077	7.812
Separation of rainwater and sewage (***C***_8_)	6	8	7	10	6	10	6.835	8.806	7.696	0.971	7.608
Outdoor ventilator/air cleaning device (***C***_9_)	5.3	8	7	10	6	9	6.425	8.566	7.469	1.141	7.499
Natural water characteristics (***C***_10_)	5.5	8	7.5	10	6	9	6.978	9.277	8.091	1.799	7.817
High-quality plants (***C***_11_)	6	8	8	10	7	9	6.915	9.366	8.097	2.452	8.000
Interactive leisure landscape (***C***_12_)	5	7.5	7	10	6	9	6.652	8.950	7.796	1.797	7.348
Suitable soundscape (***C***_13_)	5	7	6	10	6	9	5.996	8.050	7.062	1.054	7.344
Access to fruits and vegetables (***C***_14_)	4	7	6	10	5	9	5.643	7.803	6.697	1.160	6.571
Beautiful outdoor landscape (***C***_15_)	5	8.6	8	10	7	9	7.011	9.364	8.094	1.753	8.277
No noise pollution (***C***_16_)	5	9	8	10	6	9	6.556	8.895	7.551	1.339	8.268
Security management (***C***_17_)	6	9	8	10	7	9	7.026	9.382	7.975	1.356	8.412
Gathering social square (***C***_18_)	4	8	7	9.6	5	8	5.611	7.769	6.517	1.158	7.243
Open multifunctional spaces (***C***_19_)	6	9	8	10	7	9	6.826	9.004	7.859	1.178	8.316
Optimization of children's playground (***C***_20_)	5	8	7	10	6	9	5.822	7.844	6.767	1.021	7.279
Pet activity space (***C***_21_)	5	7	6	10	5	8	5.595	7.613	6.621	1.019	6.534
Preservation of community culture (***C***_22_)	5	9	7	10	6	9	6.954	8.981	7.884	0.026	7.984
Public health service station (***C***_23_)	6	9	8.7	10	7	9	7.373	9.638	8.308	1.965	8.810
Number of evaluation elements selected				21						Threshold value	6.667

**Table 3:** Performance Evaluation of the Case Study using VIKOR.

Dimensions/ Criteria	Influential Weights (IWs)	Old Nanhaixian Community		Sanfu Community		Panqiu Community	
		Performance f1j	Gap Ratio	Performance f2j	Gap Ratio	Performance f3j	Gap Ratio
Outdoor Activities(*D*1)	**0.337**	**3.287**	**0.343**	**2.703**	**0.460**	**3.161**	**0.368**
Friendly slow walking (*C*1)	0.134	4.136	0.173	2.700	0.460	3.350	0.330
Public transport connectivity and accessibility (*C*2)	0.097	3.818	0.236	3.350	0.330	3.600	0.280
Readable guidance systems and hazard indicators (*C*3)	0.097	3.591	0.282	2.900	0.420	3.250	0.350
Suitable lighting (*C*4)	0.089	3.364	0.327	2.850	0.430	3.150	0.370
Adequate seating (*C*5)	0.171	3.091	0.382	2.700	0.460	3.350	0.330
Convenient infrastructure (*C*6)	0.118	2.818	0.436	2.350	0.530	2.750	0.450
Sports entertainment facilities (*C*7)	0.126	2.909	0.418	2.700	0.460	3.200	0.360
Separation of rainwater and sewage (*C*8)	0.104	2.909	0.418	2.500	0.500	2.850	0.430
Outdoor ventilator/air cleaning device (*C*9)	0.063	2.864	0.427	2.200	0.560	2.650	0.470
Touch Nature(*D*2)	**0.326**	**3.002**	**0.400**	**2.323**	**0.535**	**2.199**	**0.560**
Natural water characteristics (*C*10)	0.171	2.227	0.555	1.800	0.640	1.750	0.650
High quality plants (*C*11)	0.165	2.864	0.427	2.100	0.580	2.200	0.560
Interactive Leisure Landscape (*C*12)	0.182	2.636	0.473	2.250	0.550	2.250	0.550
Suitable Soundscape (*C*13)	0.157	3.046	0.391	2.400	0.520	2.400	0.520
Beautiful outdoor landscape (*C*15)	0.153	3.546	0.291	2.400	0.520	2.600	0.480
No noise pollution (*C*16)	0.171	3.773	0.246	3.000	0.400	2.050	0.590
Social interaction(*D*3)	**0.337**	**3.570**	**0.286**	**2.765**	**0.447**	**3.262**	**0.348**
Security management (*C*17)	0.180	4.091	0.182	3.150	0.370	3.550	0.290
Gathering social square (*C*18)	0.174	3.227	0.355	2.850	0.430	3.400	0.320
Total Performance		**3.289**		**2.600**		**2.881**	
Total Gap			**0.342**		**0.480**		**0.424**

Note: gap ratio *r*_*kj*_=(|*f*_*j*_^aspired^ − *f*_*kj*_|)/(|*f*_*j*_^aspired^ − *f*_*j*_^worst^|)=(5 − *f*_*kj*_)/(5 − 0), *s*_*k*_=∑_*j*=1_^*n*^ *w*_*j*_ *r*_*kj*_, where *f*_*kj*_ denotes *j* criterion *f* of alternative, i.e., *f*=1(*ONHX*), *f*=2(*SF*) and *f*=3(*PQ*).

## Data Availability

The data used to support the findings of this study are included within the article.
